# Clinical Impact Potential of Supplemental Nutrients as Adjuncts of Therapy in High-Risk COVID-19 for Obese Patients

**DOI:** 10.3389/fnut.2020.580504

**Published:** 2020-10-22

**Authors:** Emre Sahin, Cemal Orhan, Fatih M. Uckun, Kazim Sahin

**Affiliations:** ^1^Department of Nutrition, School of Veterinary Medicine, Firat University, Elazig, Turkey; ^2^COVID-19 Task Force, Reven Pharmaceuticals, Golden, CO, United States; ^3^Department of Developmental Therapeutics, Immunology and Integrative Medicine, Ares Pharmaceuticals, St. Paul, MN, United States

**Keywords:** SARS-CoV-2, inflammation, cytokines, micronutrients, nutrition

## Abstract

The emergence of severe acute respiratory syndrome coronavirus 2 (SARS-CoV-2) disease (COVID-19) in China at the end of 2019 caused a major global pandemic and continues to be an unresolved global health crisis. The supportive care interventions for reducing the severity of symptoms along with participation in clinical trials of investigational treatments are the mainstay of COVID-19 management because there is no effective standard therapy for COVID-19. The comorbidity of COVID-19 rises in obese patients. Micronutrients may boost the host immunity against viral infections, including COVID-19. In this review, we discuss the clinical impact potential of supplemental nutrients as adjuncts of therapy in high-risk COVID-19 for obese patients.

## Introduction

Severe acute respiratory syndrome coronavirus 2 (SARS-CoV-2) disease (COVID-19) has been declared a pandemic by the World Health Organization with more than 13.378.800 million confirmed cases and more than 580.000 deaths worldwide (1). Immunocompromised risk groups of the populations have high mortality rates because of the insufficient host immunity (2, 3). SARS-CoV-2 damages the respiratory tract and causes acute lung injury (ALI) (2, 4, 5). ALI triggers an inflammatory response while stimulating the immune system. This inflammatory immune response is associated with a cytokine storm that may result in a potentially fatal acute respiratory distress syndrome (ARDS) characterized by increased production of reactive oxygen species (ROS) as well as pro-inflammatory cytokines and chemokines (6). The cytokine storm may disrupt an effective anti-viral immune response and cause severe lymphocytopenia as well as T-cell exhaustion in affected COVID-19 patients (7, 8).

The nutritional status of the human body plays a pivotal role in developing an effective and appropriately balanced immune response to pathogenic viruses (9). Recent studies confirmed the importance of host nutritional status in surviving the COVID-19 challenge (6, 10, 11). The protein-energy malnutrition (PEM) causes an imbalanced immune response to viral pathogens that can result in infiltration of the lungs by inflammatory cells and the development of pneumonitis following viral infection (12). The comorbidities of COVID-19 patients are correlated with the severity of PEM and contribute to a higher risk of ARDS and increased case mortality rate (13). In COVID-19, the decreased serum albumin (14) and prealbumin (15) levels have prognostic value. Low serum prealbumin levels serve as a surrogate marker for malnutrition and a poor prognostic factor (16). We believe that the nutritional status of all COVID-19 patients should be carefully evaluated, and consideration given to patient-tailored special diet programs that ensure an adequate and balanced intake of proteins, calories, and micronutrients (17). Adequate daily protein, especially whey and soy, intake have beneficial effects on the antioxidant defense system and host immunity (18). Among the various sources of protein, the whey protein has been recently recommended as a well-balanced and easy to digest amino acid and protein source with anabolic (19), anti-inflammatory (20), and immunomodulatory properties (21) as well as antiviral effects (22). Besides being an energy-rich part of the daily diet for balanced caloric intake, dietary fats, including fish oil and vegetable oils, provide a source for essential fatty acids as well as fat-soluble vitamins affecting metabolism and immunity. The essential fatty acid alpha-linolenic acid (ALA, 18:3n-3, omega-3), and the semi-essential fatty acids eicosapentaenoic acid (EPA), and docosahexaenoic acid (DHA) (23, 24), can be useful in supporting immune defense and the treatment of inflammatory diseases caused by both viruses and bacteria (25). In addition, essential micronutrients including vitamins and minerals, play an important role for the functional integrity and responsiveness of our immune system. Some of the vitamins (A, pyridoxine, cobalamin, folate, C, D, and E) and trace minerals such as Zn, Cu, Se, and Fe takes the crucial role to maintain and support the immune system (6). Balanced nutrition and intake of nutrients in appropriate amounts and composition may reduce the levels of pro-inflammatory cytokines and their side effects in COVID-19 patients (10).

Obesity has a rising prevalence, and it is considered a clinically significant risk factor for metabolic diseases as well as infections (26, 27). The consumption of “poor quality” foods often results in a nutritional deficiency in obese persons despite the higher than average amounts of food consumed (25). Such a nutritional deficiency may increase the severity of COVID-19 with increased morbidity and mortality (28). In this review, we discuss the possible role of micronutrients in the pathophysiology and survival outcome of COVID-19. We also review the current knowledge about the emerging role of supplemental nutrients as adjuncts to the supportive care for COVID-19, especially in obese patients.

## Obesity and COVID-19

**Figure 1:**
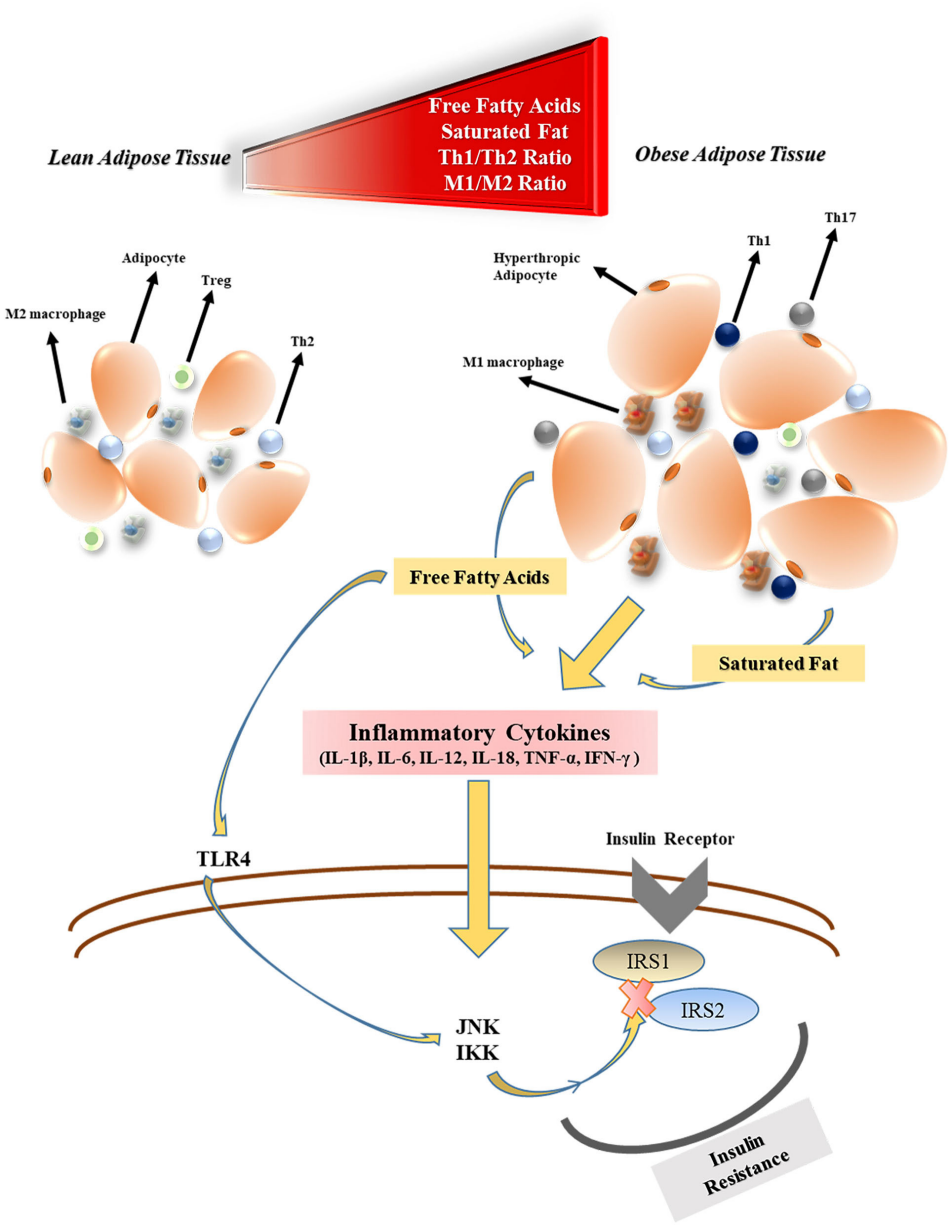
The effects of obesity on inflammatory cytokine production and insulin resistance. Treg, regulatory T cell; Th2, T helper cell 2; M1, type 1 macrophages; FFA, free fatty acid; TLR4, toll-like receptor-4; JNK1, c-Jun N-Terminal Protein Kinase 1; IKK, Iκβ kinase; IRS, insulin receptor substrate.

Obesity has detrimental effects on pulmonary function. Functional residual capacity and expiratory reserve volume are negatively affected by obesity as a consequence of the airway closure by fat accumulation in the mediastinal, thoracic, and abdominal cavity (29). Along with the rising body fat content, excessive secretion of adipokines, and cytokines from adipose tissue are thought to both compromises the immune system responsiveness to infections and cause systemic inflammation (27). The levels of free fatty acid (FFA), and lipopolysaccharide (LPS) released by gut bacteria increase during obesity which triggers activation of the (i) Toll-like receptor 4 (TLR4) pathway, (ii) adipose tissue macrophages (30) as well as (iii) nuclear factor-kappa β (NF-κβ) pathway (31). M1 phenotype macrophages that initiate and regulate inflammatory reactions through interferon-gamma (IFN-γ), TLR4, LPS, and FFA stimulation become the primary immune system elements located in the adipose tissue (32). Many inflammatory cytokines such as tumor necrosis factor-alpha (TNF-α), interleukin-1β (IL-1β), IL-6, IL-12, and IL-18 are secreted by M1 macrophages. Therefore, T helper cell 1 (Th1) to Th2 ratio, (26) and Th17 increase in obesity, which results in an immune imbalance (33). M1 macrophages also play an essential role in the development of tissue-level insulin resistance in obesity. This is owing in part to the elevated levels of inflammatory factors that impair the c-Jun N-Terminal Protein Kinase 1 and Iκβ kinase/NF-κβ cascades which regulate phosphorylation of insulin receptor substrates (IRS1 and IRS2) (34) (Figure 1). Besides the insulin resistance, the beta-cell function of the pancreas may be decreased during longterm obesity due to the continuous FFA exposure that activates the NF-κβ signaling pathway (35). In addition, venous thromboembolism is encountered more frequently by obese individuals due to the prothrombotic effects of low-grade chronic inflammation (36–38). The prothrombotic effects of inflammation are triggered by platelet activation (39), increased activity of coagulant factors (factor VIIa, VIII, IX, X, fibrinogen, and von Willebrand factor), stimulation adhesion molecules (P-selectin), and downregulation of endogenous anticoagulant factors (antithrombin, and protein C) (40). In addition, the plasminogen activator inhibitor-1 (PAI-1), a prothrombotic adipokine, also contributes to augmented venous thromboembolism of obesity (41). Coagulopathy, including DIC, is one of the main causes of mortality in COVID-19 (42).

**Figure 2:**
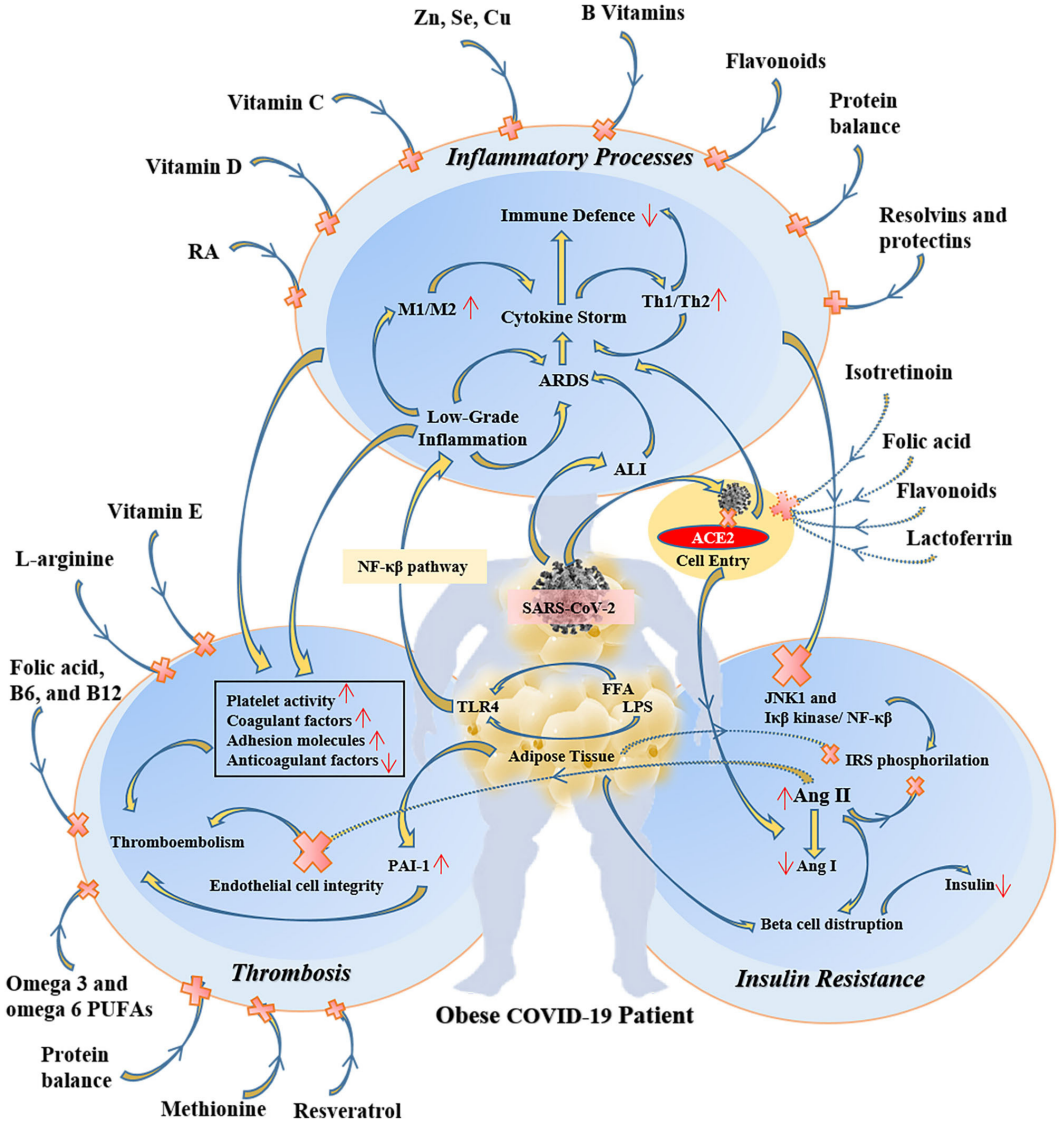
The protective and therapeutic effects of some specific micronutrients on inflammatory, thrombotic, and insulin resistance pathways in obese COVID-19 patients. RA, retinoic acid; M1, type 1 macrophages; Th1, T helper cell 1; ARDS, acute respiratory distress syndrome; ALI, acute lung injury; ACE2, angiotensin-converting enzyme 2; NF-κβ, nuclear factor-kappa β; LPS, lipopolysaccharide; FFA, free fatty acid; TLR4, toll-like receptor-4; JNK1, c-Jun N-Terminal Protein Kinase 1; IRS, insulin receptor substrate; Ang, angiotensin; PAI-1, plasminogen activator inhibitor-1.

Because of the overlapping systemic inflammation of obesity and systemic inflammation triggered by viral sepsis in COVID-19, obese patients with COVID-19 experience greater severity of pulmonary and metabolic complications as well as multi-organ dysfunction (43–45). Therefore, several micronutrients may have clinically meaningful beneficial effects in obese COVID-19 patients (46) (Figure 2). In COVID-19 patients many evidence demonstrates the metabolic link between inflammatory state and cytokine storm that mainly responsible for respiratory symptoms (45).

Several studies have shown a relationship between high BMI and severity of COVID-19. Ho et al. (47) reported that the risk of critical illness in COVID-19 increases by 44% for overweight people and almost doubles for obese patients. Likewise, an observational study using electronic health records indicated that obesity is an important contributing factor for case mortality in COVID-19 (48). Because obese patients have an impaired immune system, they may have inadequate cellular immune responses to pathogens and this acquired immunodeficiency increases their susceptibility to infections (49). In addition to the reduced T-effector cell function, the unbalanced production of immunomodulatory endocrine hormones also contribute to poor host immunity of obese persons against infections (50). Balanced nutrition and micronutrients help prevent the unfavorable outcomes observed in both obesity and COVID-19 (Table 1) (51–78).

**Table 1:** The possible effects of some micronutrients on common symptoms of COVID-19 and obesity.

**Micronutrients**	**Key Mechanism of Action**	**Outcomes**	**References**
Vitamin A	• Inhibition of M1 macrophage • Downregulation of IFN-γ • Promotion of Treg via inhibiting Th17 shifting • Inhibition of ACE2 by isotretinoin • Epithelial cell repairing properties	• Inflammatory status ↓ • Probably insulin resistance ↓ • Probably viral replication ↓ • Lung damage prevention	(51–55)
Vitamin C	• Protection of the respiratory system • Promotion of antioxidation and anti-inflammation properties • ROS scavenging activity • Inhibition of NF-κβ pathway	• Inflammatory status ↓ • Immunity ↑	(56)
Vitamin D	• Reduction of the risk of contracting respiratory infections • Regulation of Th1/Th2 balance	Immunity ↑	(57)
Vitamin E	• ROS scavenging activity • Inhibition of acute neutrophil inflammation in lung	• Inflammatory status ↓ • Lung damage prevention	(58, 59)
B vitamins	• Protection the respiratory system • Reduction of the risks of infection and re-infection • Reduction of inflammatory cytokine production • Regulation of the CD4/CD8 ratio and natural killer cell activity by vitamin B12 • Inhibition of ACE2 by folic acid • Prevention of hyperhomocysteinemia by folic acid, B6, and B12	• Inflammatory status↓ • Lung damage prevention • Immunity ↑ • Probably insulin resistance ↓ • Probably viral replication ↓ • Prevents thromboembolism	(60–64)
Selenium	• Antioxidative and anti-inflammatory properties in high-risk adults • Regulation of M1/M2 macrophage • Cofactor for glutathione peroxidase • Oxidizing capacity • Anticoagulant	• Inflammatory status ↓ • Antiviral activity ↑ • Prevents thromboembolism	(65, 66)
Zinc	• Antioxidative and anti-inflammatory properties in high-risk adults • Reduction of inflammatory cytokine production • Regulation of Th1/Th2 balance • Inhibition of ACE2 • Exert antiviral effect	• Inflammatory status↓ • Immunity ↑ • Probably insulin resistance ↓ • Probably viral replication ↓	(67–73)
Copper	• Regulation of Th1/Th2 balance • Reduction of inflammatory cytokine production • Oxidizing activity	• Inflammatory status↓ • Antiviral activity ↑	(74–76)
Magnesium	• Reduction of inflammatory cytokine production • Regulation of M1/M2 macrophage • Inhibits PARP	• Inflammatory status↓ • Prevents thromboembolism • Lung damage prevention	(77, 78)

The complex interplay between obesity and COVID-19 is explained by several mechanisms. SARS-CoV-2 uses the angiotensin-converting enzyme-2 (ACE2) for cell entry, and the amount of this transmembrane enzyme is found larger amounts in obese individuals (79). It is probably due to higher ACE2 expression in adipocytes of people with obesity (80). Therefore, adipose tissue of obese individuals can be a potential target for SARS-CoV-2 before spreading to other organs (81). Assuming that SARS-CoV/CoV-2 affects the pulmonary lipofibroblast transcriptional program that leads to pulmonary fibrosis, the use of peroxisome proliferator-activated receptor-gamma (PPARγ) agonists could be an option to reduce the risk of pulmonary fibrosis. This strategy may show strong anti-fibrotic effects that disrupt myofibroblast differentiation and transforming growth factor-beta (TGF-β) signaling. PPARy induction could lead to an effective reduction of the problem and organ fibrotic disease, including pulmonary fibrosis (81) by decreasing the fat mass of adiponectin (82).

The Spike glycoprotein (S), a structural protein of SARS-CoV-2, is responsible for binding to the host cell. Hoffmann et al. (83) reported that S protein is primed serine protease and recognized by the cell receptor. Liu et al. (84) showed that the S protein of SARS-CoV-2 had two trimers that bind to the ACE2 heterodimer. Dipeptidyl peptidase 4 (DPP4) is a ubiquitous membrane-bound aminopeptidase that circulates in the plasma has multifunctional roles in metabolism, immunity, and the endocrine system. DPP4 regulates glucose homeostasis and inflammation differently through its immunomodulatory properties. Bassendine et al. (85) reported that obesity and metabolic syndrome strongly affect the severity of COVID-19 by modulating the DPP4 expression.

In COVID-19, loss of the ACE2 enzyme that converts angiotensin-II to angiotensin-I may lead to insulin resistance (86) and endothelial cell dysfunction (87). Although the lung tissue is the primary target of SARS-CoV-2, ACE2 protein expression levels in adipose tissue (80) and pancreas (88) are higher than in the lung. High-level ACE2 expression in the pancreas may predispose to viral pancreatitis (88) and subsequently diabetes as a complication. Therefore, obese individuals may be at higher risk for metabolic complications of COVID-19.

## Micronutrients and COVID-19 Patients With Obesity

The insufficiency of micronutrients including vitamin A, vitamin D, vitamin E, vitamin B1, vitamin B6, vitamin B12, vitamin C, Fe, Zn, and Se, called “latent hunger” causes important health problems globally. Correa-Rodríguez et al. (89) observed significant reductions in vitamin C, vitamin A, and Se intake in overweight or obese young adults. Therefore, the supplementation of micronutrients may support the body's natural defense system by enhancing immunity, epithelial barriers, cellular immunity, and antibody production (90).

### Vitamins

#### Vitamin A

Vitamin A is accepted as an anti-inflammatory or anti-infective micronutrient owing to its immunomodulatory, and epithelial cell repairing functions (55). Vitamin A has been shown to reduce the severity of viral pneumonia caused by an avian coronavirus (91), measles (92), influenza A, rotavirus, and Newcastle disease virus (93). Therefore, retinoids could potentially inhibit the replication of SARS-CoV-2 and thereby reduce the severity of COVID-19 (94). Furthermore, the vitamin A derivate named isotretinoin (54) may interfere with the cellular uptake of SARS-CoV-2 and its lung-directed pathogenicity by inhibiting the ACE2 (95). Administration of all-trans retinoic acid in a hypoxia/reoxygenation model increased the mRNA expression of ACE2 and down-regulated the mRNA expression of ACE1 and TGF-β1 in renal tubular epithelial cells (96). In individuals with low vitamin A levels, histopathological changes have been detected in pulmonary epithelia and lung parenchyma, along with an increased risk of pulmonary dysfunction and respiratory disease (97). Normal serum retinol levels may mask the severity of vitamin A deficiency in obesity (98). The continuous consumption of the western diet reduces tissue vitamin A levels (99). Vitamin A deficiency and increased expression of leptin, enhance the levels of pro-inflammatory cytokines that contribute to the systemic inflammation in obesity (100). Penkert et al. (98) reported that vitamin A supplementation protects against a respiratory virus infection by controlling respiratory virus clearance, decreasing inflammatory cytokines in the blood, and altering the lung immune capacity in obese C57BL/6 mice. In addition, high doses of oral vitamin A supplementation has been shown to reduce obesity by upregulating brown adipose tissue-uncoupling protein1 (BAT-UCP1) expression in the WNIN/Ob rat model (101). Considering the effects of COVID-19 on lung function and protective properties of vitamin A in the organism, vitamin A is expected to have a beneficial effect in obese COVID-19 patients.

#### Vitamin C

Vitamin C has been used to boost the antiviral host immune defense (56), reduce or prevent the symptoms of the common cold and other respiratory infections caused by viruses (102, 103). Vitamin C regulates the immune responses in the early stage of influenza infection through increasing the levels of type I interferons (IFN-α and IFN-β) (104) having critical functions to attenuate viral pathogenesis (105). Vitamin C is an effective intracellular antioxidant for biomolecules and has significant ROS scavenging activity that results in the inhibition of the inflammatory NF-κβ signal transduction pathway (56). In addition, the phagocytic activity of neutrophils and macrophages is regulated by their vitamin C content (106). Supplemental vitamin C may decrease the severity of obesity and its co-morbidities by regulating lipid accumulation, inhibiting lipolysis that reduces systematic FFA efflux, and glucocorticoid production, reducing ROS activity and interfering adipocyte macrophages, thus decrease pro-inflammatory adipokines (leptin) and cytokines (107). In a meta-analysis of eight randomized clinical trials in 3,135 children aged 3 months to 18 years, vitamin C administration decreased to the duration of upper respiratory tract infection by 1.6 days. In the same study, it was reported that children 6 years of age benefit from more effective vitamin C administration associated with Echinacea (108). In a randomized, double-blind, placebo-controlled, phase I trial, ascorbic acid infusion rapidly increased plasma ascorbic acid concentration and reduced the pro-inflammatory biomarkers C-reactive protein (CRP) and procalcitonin levels, prevented an increase in thrombomodulin levels consistent with reduced vascular damage, and caused reductions in sequential organ failure assessment scores (109). In addition, a time-delayed infusion protocol of both ascorbic acid and dehydroascorbic acid attenuated pro-inflammatory, procoagulant states that induce lung vascular damage and significantly prolonged survival (110). In obesity, low vitamin C status correlates with inflammatory reactions and vascular dysfunction (111), and a dose of 1 g/day vitamin C treatment for 8 weeks could reduce CRP and IL-6 levels in both hypertensive and diabetic obese patients (112). In higher doses, vitamin C can act as an oxidizing agent (106). The oxidizing properties of vitamin C are boosted with the presence of iron, increasing its antiviral activity via the Fenton reaction that results in the production of hydrogen peroxide and hydroxyl radicals (113). The different doses of vitamin C supplementation (125 and 250 mg/kg) reduced mitochondrial antiviral signaling, interferon-regulating factor 3, and steroid hydroxylase in mice exposed to restraint stress and H1N1-induced pneumonia (114). Recently, Peng (115) started a randomized controlled vitamin C infusion trial that aims to attenuate the respiratory symptoms of COVID-19 infection. High-dose vitamin C might be an effective choice in the early treatment of COVID-19 (116). Consumption of citrus fruits and vegetables containing vitamin C has been proposed as a low-cost strategy to support the immune system during the COVID-19 pandemic (11).

#### Vitamin D

**Figure 3:**
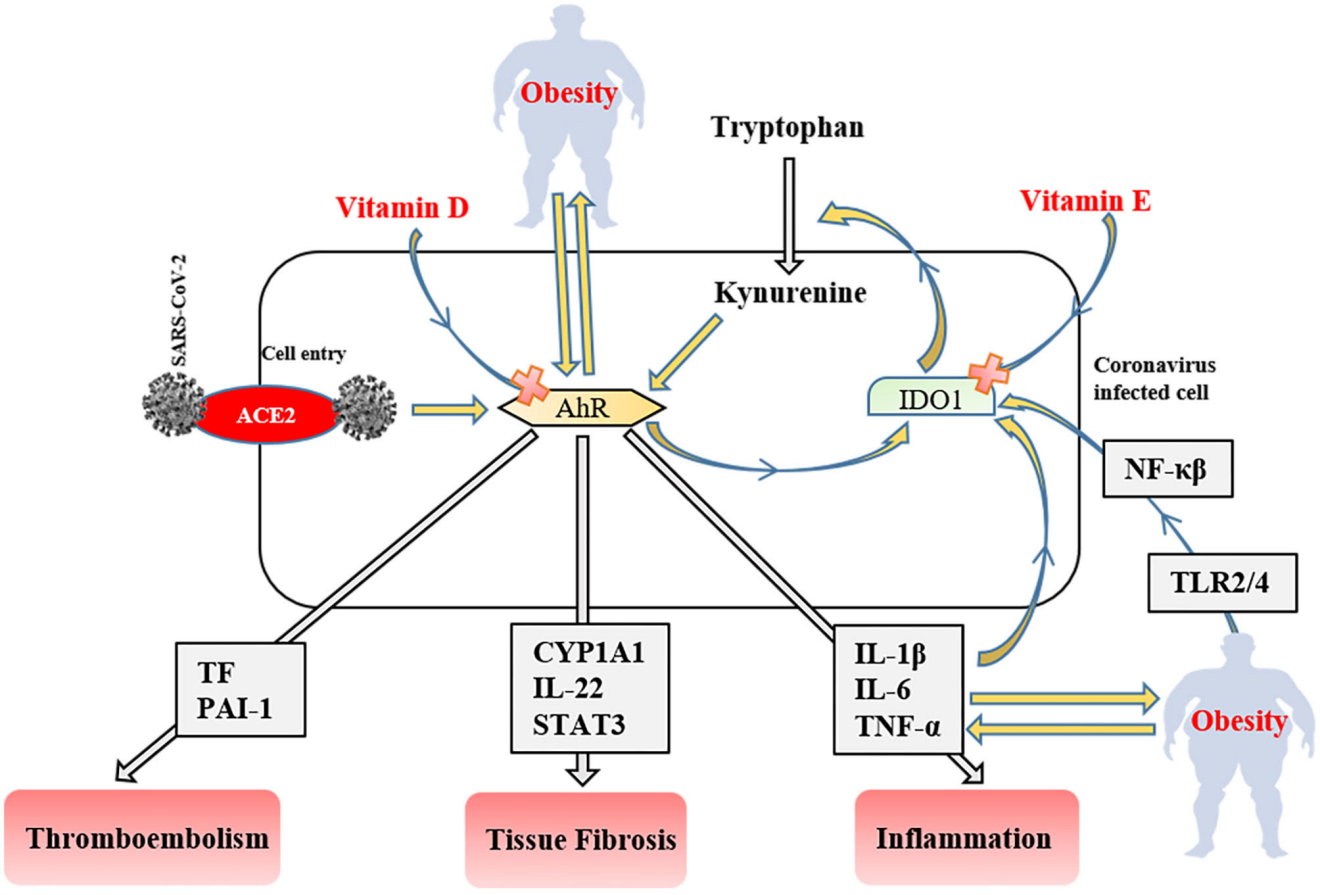
The role of the IDO1-AhR-IDO1 signaling loop in obese COVID-19 patients. AhR, aryl hydrocarbon receptors; IDO1, indoleamine 2,3-dioxygenase; ACE2, angiotensin-converting enzyme 2; NF-κβ, nuclear factor-kappa β; TLR, tool-like receptor; PAI-1, plasminogen activator inhibitor-1; CYP1A1, Cytochrome P450, family 1, subfamily A, polypeptide 1; STAT3, signal transducer and activator of transcription 3; TNF-α, tumor necrosis factor-alpha IL; interleukin TF, tissue factor.

There are multiple variables such as age, body mass index, skin color, and genetic variants that can affect the vitamin D stores of the body (117). Low serum 25-hydroxyvitamin D [25(OH)D] concentrations have been reported in obese humans and an inverse relationship between BMI and serum 25(OH)D has been reported in obese humans (118). Lin et al. (119) were observed that vitamin D deficiency [25(OH)D <20 ng/mL] and insufficiency [20 <25(OH)D <30 ng/mL] in 52 obese (mean BMI 37.6 ± 6.4 kg/m^2^) were at 73 and 22% prevalence. Vitamin D metabolizing enzyme expression (Cyp2r1, Cyp27a1, and Cyp2j3) was affected by high fat diet-induced obesity, which may partially explain the mechanisms of the modified vitamin D endocrine system related to obesity (120). Adequate daily intake of vitamin D is thought to curb viral infections (121, 122). Seasonal viral infections affecting the respiratory tract as well as COVID-19 may be facilitated by vitamin D deficiency (123, 124). The serum levels of 25(OH)D, the circulating metabolite of vitamin D, are inversely correlated with pulmonary inflammation (125) and directly correlated with the pulmonary function (120) as well as host immune response (126) during respiratory virus infection. 1,25-dihydroxyvitamin D (1,25D), an active metabolite of vitamin D, has pleiotropic effects on immune system elements (57, 127–129) and may reduce the production of pro-inflammatory cytokines that have been implicated in the pathophysiology of COVID-19 associated ARDS (IFN-γ, TNF-α, IL-1, IL-6, IL-2, IL-12, and IL-17) (Figure 3) (130). Administration of vitamin D stimulated binding of the SARS-CoV-2 cell entry receptor ACE2 to angiotensin-II receptor type 1, decreasing the number of virus particles that could attach to ACE2 and enter the cell (131, 132). Therefore, vitamin D supplementation could potentially reduce the incidence of severe COVID-19 (124). In randomized controlled trials of vitamin D for prevention of respiratory tract infection (Of 1,137 citations retrieved, 11 placebo-controlled studies of 5,660 patients), vitamin D showed a protective effect against respiratory tract infection, with daily dosing appearing to be the most effective strategy (133). That being said, a clinical study conducted by Hastie et al. (134) indicated that the relation between COVID-19 and serum vitamin D levels was not significant. Additionally, the intake of high doses of vitamin D may have harmful effects on COVID-19 patients (124).

#### Vitamin E

Vitamin E supplementation generally reduces the severity of infectious diseases, owing to antioxidant and immunomodulatory effects (135). Tocopherols (α and γ), natural vitamin E isomers, exhibits ROS scavenging activity (58) and can block acute neutrophil inflammation in the lung (59). Dietary vitamin E deficiency may increase IL-1 induced lung leak in rats (136), and probably provokes acute hyperoxic lung injury related to IL-6 and 8-iso-prostaglandin F_2α_ stimulated inflammation in mice (137). Oral vitamin E intake is positively associated with lung health (138). In a randomized clinical trial performed by Agler et al. (139) proved that a dose of 600 IU vitamin E (every other day) reduced the risk of chronic lung disease. No studies have been done in the case of the link between vitamin E and COVID-19 patients with obesity. Meydani et al. (140) found that a daily intake of 200 IU vitamin E has not any effect on lower respiratory infection. In the same study, the incidence of upper respiratory infections such as common cold found lower in older people.

After ACE2 mediated cell entry, coronaviruses firstly activate aryl hydrocarbon receptors (AhRs) without indoleamine 2,3-dioxygenase (IDO1) stimulation (141). Activated AhRs initiates the production of inflammation factors (IL-1β, IL-6, and TNF-α), induces tissue factor (TF) and PAI-1 mediated thromboembolism (AhR-TF/PAI-1 pathway) (142), and lead multiple organ fibrosis via Cytochrome P450, family 1, subfamily A, polypeptide 1 (CYP1A1)/IL-22 signaling pathway with signal transducer and activator of transcription 3 (STAT3) (143). The produced inflammatory cytokines trigger IDO1 that metabolizes tryptophan to kynurenine (AhR stimulator). Consequently, these signaling factors lead to the IDO1-AhR-IDO1 loop (Figure 3) (141). AhR signaling pathway stimulation also modulates obesity via disrupting fat metabolism (144). The degree of AhR activation raises depend on the severity of obesity due to enhanced inflammatory factors, including TLR2/4- NF-κβ mediated (145). Likewise, elevated dietary fat intake increases the level of serum (low-density-lipoprotein) LDL (146) that responsible for the AhR activation (147). SARS-CoV-2 induced IDO1-AhR-IDO1 loop might be exponentially increased in obese COVID-19 patients, and probably inhibited by vitamin D for AhR (148), and vitamin E for IDO1 (149). Therefore, using both vitamin D and E together most likely reduces the clinical symptoms in COVID-19 infection and obesity-associated complications.

#### B Vitamins

The prevention or treatment of lung damage is critical for the survival outcome of COVID-19 patients. Vitamin B3, a claimed protector of lungs, can promote the healing of tissue damage in the lungs (60, 61), most likely owing to its ability to inhibit the poly ADP ribose polymerase (PARP) (61). Because the increased activity of PARP elevates the inflammatory cytokines that contribute to the cytokine storm, vitamin B3 therapy may reduce cytokine storm in COVID-19 (6). The pyridine-nucleoside form of vitamin B3 called nicotinamide riboside functions as a precursor to nicotinamide adenine dinucleotide (NAD^+^), prevented ALI/ARDS and heart injury, and improved the survival of mice after the LPS challenge or sepsis caused by intraperitoneal injection of feces (150). In LPS-challenged rats, the administration of nicotinamide prevented the decrease in mitochondrial respiration and intracellular NAD^+^ levels in macrophages (151). In obesity, adipose tissue nicotinamide phosphoribosyltransferase (NAMPT) expression reduces, and NAD^+^ biosynthesis impairs. The reduction in NAD^+^ levels causes decreasing adiponectin and increasing FFA production (152). The dietary supplementation of NAD^+^ precursors alleviates inflammation, improves insulin sensitivity, and reduces body weight gain in obesity (153).

Vitamin B6 levels and the severity of inflammatory reactions are inversely correlated (154, 155). The utilization of vitamin B6 rapidly increases under inflammatory conditions, and COVID-19 patients probably may have vitamin B6 deficiency (156). Vitamin B6 downregulated the pulmonary inflammation by inhibiting macrophage activation, as reduced production of IL-1β, IL-6, and TNF-α in macrophages challenged with LPS of mice (155). In a rat model of systemic inflammation, orally administered 500 mg/kg riboflavin and 600 mg/kg thiamine increased the anti-inflammatory activity of dexamethasone, along with reducing TNF-α and IL-6 production (157). The relationship between dexamethasone and thiamine caused more inhibition of IL-6 production compared to dexamethasone-induced. Multivitamin supplementation within 48 h of hospital admission, including thiamine, riboflavin, and niacin was associated with lower overall mortality in patients with Ebola Virus Disease (158).

The folic acid and its derivates such as tetrahydrofolic acid and 5-methyl tetrahydrofolate may interfere with the cell entry of SARS-CoV 2 (63) via inhibition of furin protein that has essential for COVID-19 progression (159). In addition to the ACE2 protein, S-glycoprotein, some of the proteases (M^pro^ and PL^pro^), RNA dependent RNA polymerase, and Nsp15 promote the cellular entry of SARS-CoV 2 (63, 160). In a virtual screening study among the 106 nutraceuticals, the folic acid and folic acid derivates were identified as potential agents that could have potential in post-exposure prophylaxis (63).

The deficiency of some B vitamins (folic acid, B6, and B12) and dietary essential amino acid methionine results in hyperhomocysteinemia that leads to venous thromboembolism (64). In obesity, the high cardiovascular risk that is related to hyperhomocysteinemia correlates with insufficient nutritional status of folate and vitamin B12. The decreased levels of plasma folate and vitamin B12 are accepted as a predictor of vascular dysfunction (161). A high dose of vitamin B6 administration may reduce the TNF-α, IL-6, and D-dimer levels and improves endothelial integrity along with preventing coagulopathy in COVID-19 patients (156).

### Minerals

#### Magnesium

Magnesium can alleviate inflammatory disorders, including obesity (162) and respiratory infections (78). The M1 type macrophages producing NF-κβ depended on pro-inflammatory mediators shifts to M2 type macrophages after Mg treatment and stimulates the anti-inflammatory cytokine secretion (77). The low dietary Mg intake inversely was associated with endothelial cell dysfunction and biomarkers of systemic inflammation (163). Different forms of Mg could be used against various lung diseases (164). Li et al. (78) reported that MgSO_4_ administration reduced the PARP-1 and apoptosis-inducing factor levels in LPS induced ALI mice. Recently Tan et al. (165) observed that the administration of oral vitamin D, Mg, and vitamin B12 combination reduces the clinical deterioration in COVID-19 patients. Mg nutrition may be an effective strategy for the treatment and prevention of COVID-19 infection (166, 167). Moreover, many diseases, such as obesity that cause Mg deficiency (162), probably exacerbate the clinical symptoms of COVID-19.

#### Selenium

Selenium is considered an important antioxidant trace mineral. The severity and mortality of viral infections were inversely correlated with serum Se levels in several studies (168–170). Virulence and pathogenesis levels of viruses can be increased due to the weakened immune system after the long term intake of inadequate Se containing diets (171). The M1 type macrophages increase with Se deficiency or low Se intake (65, 172). However, high amounts of Se intake lead to shifts Th2 phenotype to the Th1 phenotype (173). Zhang et al. (174) recently reported that Chinese persons with lower hair Se content had more severe COVID-19 infections. Se supplementation may therefore have clinical utility in COVID-19 pending further confirmation of the prognostic role of Se for the survival outcome of COVID-19 patients. Fakhrolmobasheri et al. (175) reported that Se could prevent cell death caused by viral replication. In patients with ARDS, sodium selenite (1 mg for 3 days and 1 mg/d for a further 6 days) supplementation replenished Se levels and Se concentrations were positively correlated with antioxidant activity. Serum concentrations of IL-1β and IL-6 were inversely associated with serum Se concentrations. Nevertheless, there was no effect on overall survival, mechanical ventilation time, and length of stay in intensive care (176). In LPS-stressed RAW264.7 cells, LPS increased mRNA profiles of inflammatory genes, while short-time Se pretreatment reduced the LPS-induced upregulation of cyclooxygenase-2, intercellular adhesion molecule−1, IL-1β, IL-6, IL-10, nitric oxide synthase, and monocyte chemoattractant protein-1 and further increased expression of IFN-β and TNF-α (177). In the same study, LPS decreased mRNA levels of selenoprotein encoding genes, whereas increased mRNA levels of thioredoxin reductases (TXNRD1and TXNRD3) in cells. Se deficiency or overexposure impairs the selenoprotein synthesis (glutathione peroxidase and TXNRDs) that cause adipocyte dysfunction leading to various metabolic disorders. The expression of these selenoproteins is decreased in obese individuals due to their lower Se status (178). In COVID-19, selenoprotein expression may also be reduced by inflammatory factors and suppressed immune status (179). Therefore, dietary Se supplementation may help alleviate the respiratory and inflammatory clinical symptoms in obese patients suffering from COVID-19.

#### Zinc

Zinc is another important trace mineral to improve the immune functions against viral infections (180, 181). In risk groups for Zn deficiency, including aging, immune deficiency, obesity, diabetes, and atherosclerosis, low Zn status may relate to severe COVID-19 risk (182). Zn can exert its antiviral effect by suppressing viral replication, improvement of mucociliary clearance and increasing immune responses, prevention of lung injury, and regulation of antiviral and antibacterial immunity (73). Zn can provide low-cost and effective adjunctive therapy for some viral diseases, including respiratory infections (183). For example, Mossad et al. (184) reported that the duration of common cold symptoms was shortened from 7.6 to 4.4 days with zinc gluconate (containing 13.3 mg elemental Zn). *In vitr*o studies showed that Zn exhibits antiviral activity by inhibiting the SARS-CoV RNA polymerase (73). Indirect data suggest that Zn may decrease the activity of ACE2. The anti-inflammatory activity of Zn depends on NF-κB signaling pathway inhibition and modulation of Treg function, which may help reduce the risk of cytokine storm in COVID-19 (73). In addition, Zn has been revealed to be vital for respiratory epithelium, owing to antioxidant and anti-inflammatory activity (185), and also the regulation of tightly binding proteins zonula occludens-1 and Claudin-1, thereby enhancing barrier functions (186). In an *in-vitro* study was demonstrated that Zn administration (10 μm Zn preincubation) inhibited respiratory syncytial virus replication by more than 1.000-fold reduction (187). Antiviral agent chloroquine, a Zn ionosphere that is used in the treatment of COVID-19, increases the Zn transport into the cells (73, 188, 189). It has recently been proposed that the severity of COVID-19 infection could be reduced with an adequate daily intake of Zn (183). Recently, Finzi (190) reported that high dose oral supplementation of Zn salts (zinc citrate, zinc gluconate, or zinc acetate) reduced the respiratory clinical symptoms of COVID-19 patients (190). In line with this information, it was suggested that Zn could be one of the most promising micronutrients for COVID-19 prevention or treatment (191, 192). The effects of Zn on obesity and respiratory viral infections may help to treatment of COVID-19 in both obese and overweight patients.

#### Copper

Copper (Cu), a trace mineral, has an important role in host immunity against viruses, regulating inflammatory responses, and boosting the immunity of the host in many infections (193–198). Elevating the Cu levels in the lung tissue has been suggested as a strategy for treating or preventing pulmonary inflammation (199). The appropriate dietary Cu intake within normal daily limits probably increases the number of phagocytic cells, the activity of Natural killer cells, the proliferation of Th cells (200), and more importantly the Th1-stimulated production of IL-2, but not TNF-α (75). In this context, Cu is may require to maintain the balance of the Th1/Th2 profile (74). The increased pro-inflammatory cytokine TNF-α causes decreasing Cu levels in the lungs during lung infections (199). Raha et al. (201) hypothesized that Cu supplementation could protect the high-risk COVID-19 patient populations with Cu deficiency from developing ARDS.

In addition, the raising of the ROS concentration may be used to exhibit the antiviral action by Cu (76). The Cu-peroxide complexes could enhance the effectiveness of this action (202). Since ROS production properties of Cu containing surfaces, SARS-CoV and SARS-CoV-2 viruses are sensitive to Cu alloys (203, 204). However, Cu supplementation may also increase the risk of sepsis and ARDS and should not be attempted outside a well-controlled clinical trial.

## Other Important Nutrients in COVID-19

### Flavonoids

Plant-derived flavonoids having anti-inflammatory, antioxidant, and antimicrobial activities (205) also have anti-obesity and anti-diabetic potential (206). In obesity and other inflammatory disorders, dietary flavonoids could inhibit inflammatory cytokine production, leptin secretion, insulin resistance, and improve immune responses (207). Polyphenols inhibit NF-κB and activator protein-1 activates nuclear factor erythroid 2–related factor 2 (Nrf2) and improves lipid profiles via enhancing HDL-cholesterol, while the reduction in LDL-cholesterol. Therefore, the intake of high-polyphenol diets shows various antioxidant, anti-inflammatory, and dyslipidemia-reducing effects (182). Vernarelli and Lambert (208) reported that dietary flavonoid consumption was inversely correlated with the severity of obesity and serum CRP levels. The inhibition of inflammatory cytokines by flavonoids (205) in the context of pulmonary infection (209, 210) may prevent the development of or reduce the negative consequences of the cytokine storm in COVID-19. Additionally, the coagulopathy associated with COVID-19 may be alleviated by flavonoids through the reduction of endothelial TF availability (211). Both *in vitro* and *in vivo* studies indicate that flavonoids exhibit antiviral activity against respiratory tract viruses including SARS-CoV and influenza (212). In a meta-analysis performed by Somerville et al. (213), flavonoids have been shown to potentially reduce the incidence of upper respiratory tract infections caused by viruses. *In silico* virtual computational screening studies have been demonstrated that natural compounds like flavonoids may inhibit SARS-CoV-2 by binding to S proteins that have an affinity to ACE2 (214). Also, Adem et al. (215) demonstrated that flavonoids may inhibit M^pro^ used by SARS-CoV-2 for viral replication. Especially, quercetin and catechins have antiviral activity on SARS-CoV (216), and probably on SARS-CoV 2 (217, 218). In addition, curcumin (219, 220) indomethacin and resveratrol have been proposed as potential supportive care supplements against COVID-19 (221).

### Lactoferrin

Lactoferrin, which shows antimicrobial activity, has anti-inflammatory and immunomodulatory activities (222). Due to its antiviral activity, many viruses, including SARS-CoV (223), could be killed by lactoferrin (224–226). The reported antiviral action of lactoferrin against SARS and COVID-19 most probably stems from blocking the activity of ACE2 and Heparan Sulfate Proteoglycan, which are required for cell entry of SARS-CoV and SARS-CoV 2 (227). Additionally, a clinical study performed by Serrano et al. (228) indicated that bovine liposomal lactoferrin using combined with vitamin C and Zn attenuated symptoms of COVID-19 infection. Likewise, lactoferrin can alleviate obesity by inhibiting leptin production and controlling LPS releasing from gut microbiota (229). In this context, the leptin reducing functions of Zn and vitamin C (230) when combined with lactoferrin may be beneficial in the treatment of COVID-19 in obese individuals.

### Essential Fatty Acids

Dietary polyunsaturated fatty acids (PUFAs) and their metabolites exert protective effective effects during systemic inflammation (231). Supplementation of n-3 PUFAs reduces the systemic inflammation of non-diabetic obese patients (232). EPA and DHA may inhibit the inflammatory NF-κB and TLR signaling pathways (233). These long-chain fatty acids also decrease the M1/M2 macrophage ratio in adipose tissues, thereby reducing the inflammatory state and decreasing the insulin resistance (234). Omega-3 PUFAs and its lipid derivatives such as resolvins, and protectins (protectin D1), when used at appropriate dose levels and according to rational administration schedules, could be potentially useful in reducing the pro-inflammatory cytokine production that leads to cytokine storm in COVID-19 (235). Importantly, omega-3 and omega-6 PUFAs have been shown to reduce platelet aggregation and may therefore prevent thrombosis (236) and reduce the risk of thromboembolic complications in COVID-19 patients that have been associated with a poor survival outcome (237).

## Conclusion

A deficiency of micronutrients due to malnutrition has the potential to increase the severity of viral infections. Many essential nutrients like vitamins, minerals, amino acids, and fatty acids are important for the pleiotropic functions of our immune system. Balanced nutrition and intake of nutrients in appropriate amounts and composition may reduce the levels of pro-inflammatory cytokines and their side effects in COVID-19 patients. More importantly, obese COVID-19 patients are more susceptible to inflammation, lung diseases, coagulopathy, and probably insulin resistance than lean patients. Some *in vitro* and *in silico* studies suggested that specific nutrients might exhibit protective effects against COVID-19 infection. However, further studies are needed to improve our current knowledge about the emerging role of supplemental nutrients as adjuncts to the supportive care for obese COVID-19 patients.

## Author Contributions

KS and ES conceived the study, participated in its design and coordination, and drafted and authored the manuscript. CO and FU participated in the study design, interpretation of the data, and helped to draft manuscript revisions. All authors have read and approved the final manuscript.

## Conflict of Interest

FU was employed by Reven Pharmaceutcal (Golden, CO). The remaining authors declare that the research was conducted in the absence of any commercial or financial relationships that could be construed as a potential conflict of interest.
